# Network-Driven Proteogenomics Unveils an Aging-Related Imbalance in the Olfactory IκBα-NFκB p65 Complex Functionality in Tg2576 Alzheimer’s Disease Mouse Model

**DOI:** 10.3390/ijms18112260

**Published:** 2017-10-27

**Authors:** Maialen Palomino-Alonso, Mercedes Lachén-Montes, Andrea González-Morales, Karina Ausín, Alberto Pérez-Mediavilla, Joaquín Fernández-Irigoyen, Enrique Santamaría

**Affiliations:** 1 Clinical Neuroproteomics Group, Navarrabiomed, Departamento de Salud, Universidad Pública de Navarra, 31008 Pamplona, Spain; mpalomino@alumni.unav.es (M.P.-A); mercedes.lachen.montes@navarra.es (M.L.-M.); andrea.gonzalez.morales@navarra.es (A.G.-M.); jokfer@gmail.com (J.F.-I.); 2 Proteored-ISCIII, Proteomics Unit, Navarrabiomed, Departamento de Salud, Universidad Pública de Navarra, 31008 Pamplona, Spain; karina.ausin.perez@navarra.es; 3 Instituto de Investigación Sanitaria de Navarra (IdiSNA), Navarra Institute for Health Research, 31008 Pamplona, Spain; lamediav@unav.es; 4 Neurobiology of Alzheimer’s Disease, Neurosciences Division, Center for Applied Medical Research (CIMA), Department of Biochemistry, University of Navarra, 31008 Pamplona, Spain

**Keywords:** Tg2576 mice, olfactory bulb, proteogenomics, mass-spectrometry

## Abstract

Olfaction is often deregulated in Alzheimer’s disease (AD) patients, and is also impaired in transgenic Tg2576 AD mice, which overexpress the Swedish mutated form of human amyloid precursor protein (APP). However, little is known about the molecular mechanisms that accompany the neurodegeneration of olfactory structures in aged Tg2576 mice. For that, we have applied proteome- and transcriptome-wide approaches to probe molecular disturbances in the olfactory bulb (OB) dissected from aged Tg2576 mice (18 months of age) as compared to those of age matched wild-type (WT) littermates. Some over-represented biological functions were directly relevant to neuronal homeostasis and processes of learning, cognition, and behavior. In addition to the modulation of CAMP responsive element binding protein 1 (CREB1) and APP interactomes, an imbalance in the functionality of the IκBα-NFκB p65 complex was observed during the aging process in the OB of Tg2576 mice. At two months of age, the phosphorylated isoforms of olfactory IκBα and NFκB p65 were inversely regulated in transgenic mice. However, both phosphorylated proteins were increased at 6 months of age, while a specific drop in IκBα levels was detected in 18-month-old Tg2576 mice, suggesting a transient activation of NFκB in the OB of Tg2576 mice. Taken together, our data provide a metabolic map of olfactory alterations in aged Tg2576 mice, reflecting the progressive effect of APP overproduction and β-amyloid (Aβ) accumulation on the OB homeostasis in aged stages.

## 1. Introduction

Olfactory dysfunction has been related to aging and Alzheimer’s disease (AD) [1,2]. The smell impairment is considered an early event of AD, preceding the appearance of dementia symptoms. The Tg2576 transgenic mice express the hAPPSw via the hamster prion promoter, an isoform of the human amyloid precursor protein (APP) with double mutation K670N, M671L [3]. These mice displayed an increase of APP production with consequent overproduction of β-amyloid (Aβ) 40 and Aβ42 and plaques formation in the frontal, temporal, and entorhinal cortices, hippocampus, presubiculum, and cerebellum at about 11–13 months of age [4]. There is strong evidence that accumulation of Aβ peptide is responsible for age-related memory decline in these mice [5,6,7]. Other than the increase in Aβ production, these mice can also display hyperphosphorylated tau at old age. Synaptic deficits, and mitochondrial imbalance have been reported for this late-plaque model [8,9]. Metabolic and posttranslational modification alterations occur long before the onset of behavioral impairment [10,11,12]. However, Tg2576 mice did not present a profound cognitive impairment, even at old ages [13].

The olfactory bulb (OB) is the first brain structure of the olfactory pathway [14]. APP processing products have been observed in the OB of 1-month-old Tg2576 mice, as has Aβ deposition at 13.5 months of age [15]. Moreover, Tg2576 mice (between ages of 6.5 and 8 months) present a reduced rate of OB neurogenesis, a reduction in the volume of the granular cell layers of the OB [16], and some olfactory memory deficits [16,17]. A detailed analysis of olfaction in Tg2576 mice also revealed behavioral deficits in odor habituation and discrimination [18,19]. Interestingly, the appearance of these behavioral impairments corresponds with a progressive Aβ deposition in specific olfactory structures [18]. In view of these data, an in depth biochemical characterization of the OB is necessary to reveal the missing links in the biochemical understanding of smell impairments in Tg2576 AD mice.

In this study, we used a discovery platform, applying mass-spectrometry based quantitative proteomics and transcriptome-wide analyses, to decipher the pathophysiological mechanisms that are disturbed in the OB from aged Tg2576 mice (18 months of age) as compared to those of age matched background strain control mice. 107 differential genes and 25 differentially expressed proteins were detected, pinpointing specific molecular pathways, protein interactomes, and potential olfactory therapeutic targets.

## 2. Results and Discussion

OB perturbations are responsible for olfactory dysfunction in neurological syndromes [2], however, few studies have examined this structure using high throughput molecular approaches [20,21,22,23]. Focusing on the transgenic Tg2576 mouse AD model, different proteomic and transcriptomic studies were performed to characterize novel molecular mediators associated with AD pathophysiology in brain structures affected during the disease progression [9,24,25,26,27,28,29,30]. To our knowledge, this is the first study that characterizes olfactory-associated molecular changes in this late-plaque model using omics technologies.

### 2.1. Molecular Alterations Detected in the Olfactory Bulb (OB) of 18-Month-Old Tg2576 Mice

**Figure 1:**
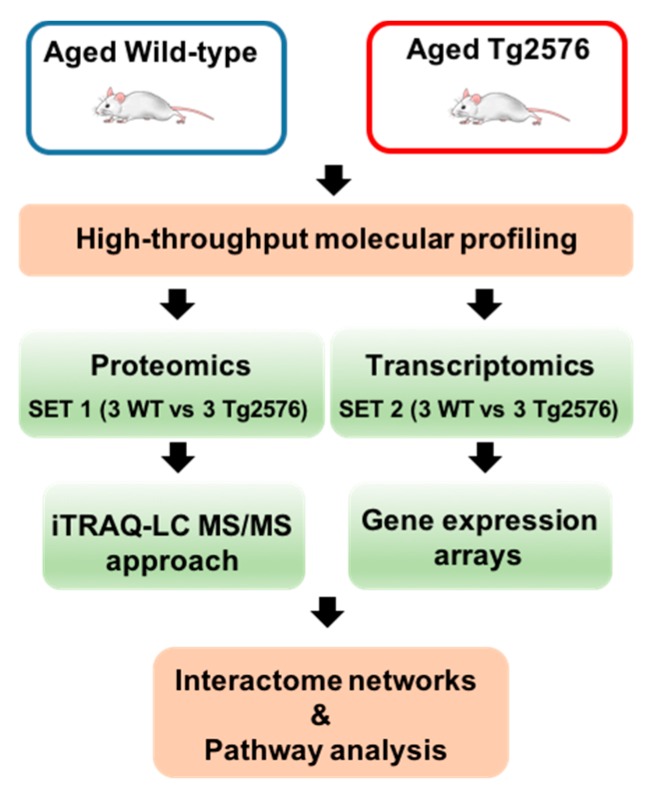
An overview of the workflow used for the molecular characterization of the olfactory bulbs (OBs) derived from aged Tg2576 mice.

Tg2576 transgenic mice suffer from memory deficits accompanied by β-amyloid plaques that increase with disease progression [5,6,7]. We have applied a dual-omic approach to analyze the molecular imbalance induced by the hAPPSw isoform at the olfactory level, with the final goal to reveal novel information about the OB site-specific molecular signature at late AD stages in 18-month-old Tg2576 mice. To analyze the potential differences in olfactory molecular expression profiles, OB specimens for each experimental group (Tg2576 and WT mice) were subjected to chemical tags (isobaric tag for relative and absolute quantitation, iTRAQ) coupled to tandem mass spectrometry (3 mice/condition) and into RNA microarray platform (3 mice/condition) (Figure 1).

**Figure 2:**
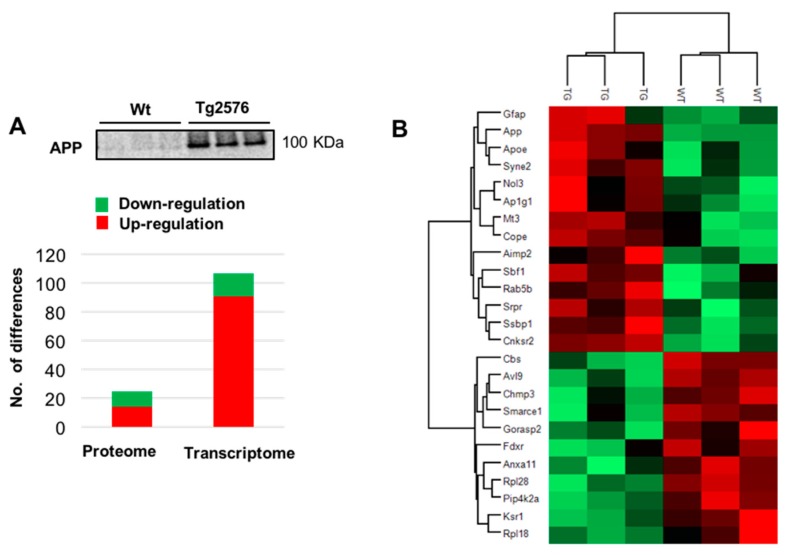

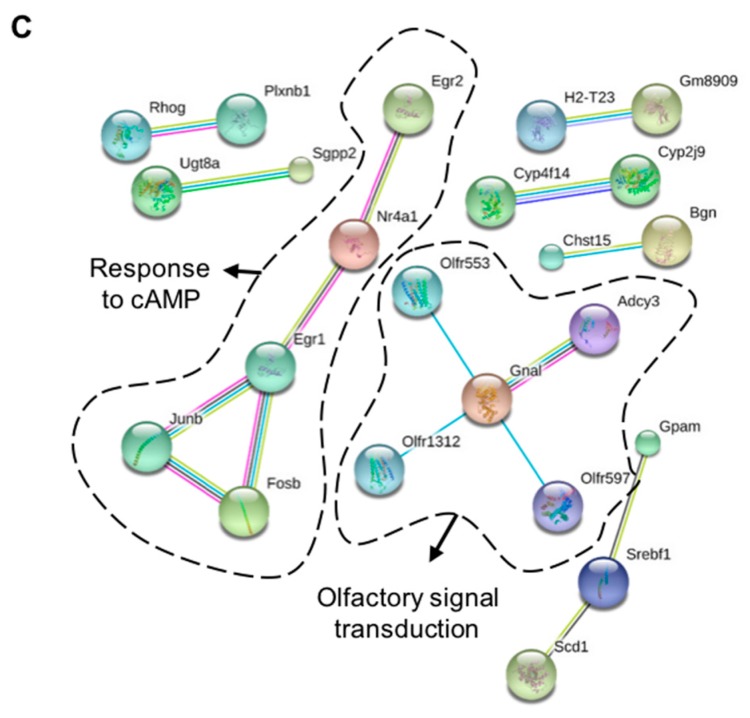
Multi-omic approach to decipher the OB site-specific molecular signature in aged Tg2576 mice. (**A**) Differential molecular profiling detected by the dual-omic approach in Tg2576 OBs. The olfactory protein expression levels of amyloid precursor protein (APP) at late Alzheimer’s disease (AD) stages in 18-month-old Tg2576 mice is shown. Equal loading control and quantitation values have been included in Table S5; (**B**) Heat map representing the degree of change for the differentially expressed proteins (Table S2) between 18-month-old wild-type (WT) and Tg2576 mice. Red and green, up- and down-regulated proteins, respectively; (**C**) Gene interactome networks for the differentially expressed genes detected in aged Tg2576 mice. Network analysis was performed submitting the corresponding gene IDs to the STRING software (v. 10.5) (Available online: https://string-db.org/). Only interactions tagged as “high confidence” (>0.7) in STRING database were considered. Dashed circles highlight the potential alteration in the response to cyclic adenosine monophosphate (cAMP) and in the olfactory transduction signaling.

Among 2466 quantified proteins (Table S1), differential analysis revealed 25 de-regulated proteins in Tg2576 OBs with respect to wild-type (WT) OBs (11 down- and 14 up-regulated in aged Tg2576 mice) (Figure 2A,B and Table S2). The up-regulation of our intrinsic positive control (APP) was verified by Western-blotting (Figure 2A). According to the STRING Database [31], this subproteome is mainly involved in membrane organization (False discovery rate (FDR): 1.21 × 10^−5^; e.g., *SYNE2*, *NOL3*, *AP1G1*), protein transport (FDR: 0.047; e.g., *RAB5B*, *RPL28*, *SRPR*, *RPL18*, *CHMP3*, *COPE*), and negative regulation of neuron differentiation (FDR: 0.041; e.g., *APP*, *APOE*, *GFAP*, *MT3*). In the transcriptomic phase, 107 protein-coding genes were differentially regulated in the OB of aged Tg2576 mice (16 down- and 91 up-regulated genes with respect to WTs) (Table S3). Gene interactome networks suggested an alteration in the response to cyclic adenosine monophosphate (cAMP) (down regulation of *EGR1*, *EGR2*, *NR4A1*, *JUNB*, and *FOSB* genes) and in the olfactory transduction signaling due to the up-regulation of *ADCY3*, and *GNAL* genes, together with the overexpression of some olfactory receptors (OR) like *OLFR553*, *OLFR1312*, and *OLFR597* genes (dashed circles in Figure 2C).

Moreover, *RTP2* (Receptor-transporter-protein 2) was also up-regulated in the OB of Tg2576 mice. RTP2 promotes OR cell-surface expression and activation in response to odorant stimulation [32]. Based on transcriptomic information from the prefrontal cortex, it has been suggested as a cause of the alteration in the smell perception of AD subjects [33]. According to our data, *OR* gene dysregulation has been also demonstrated in the OB, entorhinal, and frontal cortex in human AD subjects [34,35]. On the other hand, there was not an evident RNA-protein correlation derived from the differential datasets obtained from Tg2576 OBs. This may be due to the use of different sets of animals for each technology platform. Moreover, other reasons may also explain the observed discrepancy, such as the spatial and temporal delayed synthesis between mRNA and protein [36], post-transcriptional events, and the different hydrophobicity and solubility of the “missing proteome” during the proteomic phase, hampering its characterization and quantitation by mass-spectrometry (e.g., ORs) [37].

### 2.2. Biological Functions and Neuronal-Specific Processes Altered in the OB of Aged Tg2576 Mice

**Figure 3:**
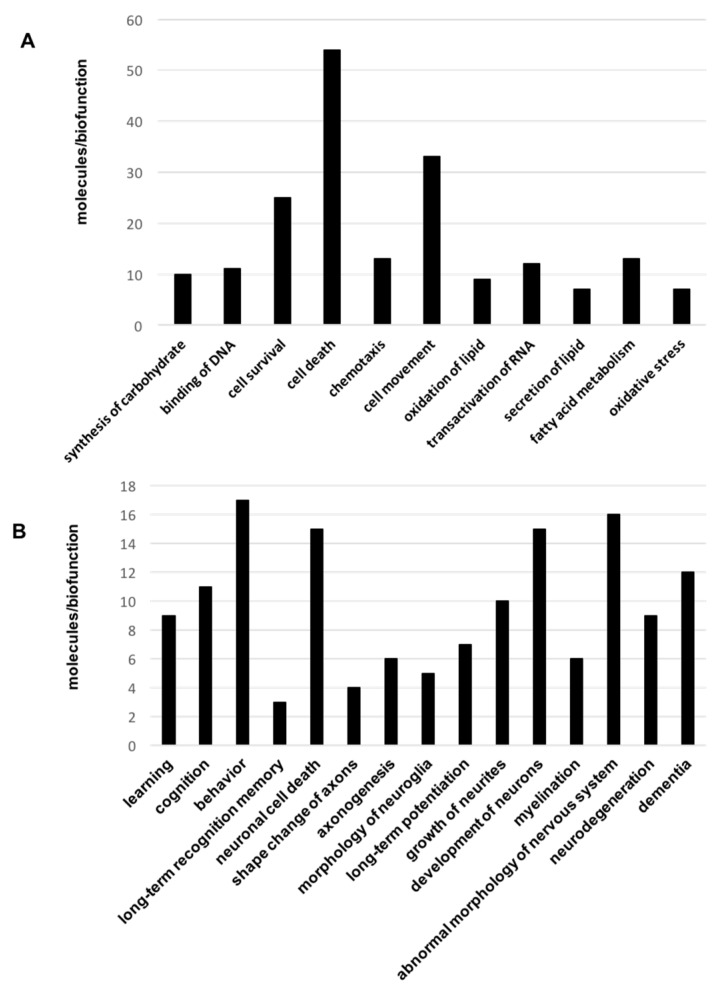
Significantly represented biofunctions in the OB of aged Tg2576 mice. Canonical (**A**) and neuronal-specific (**B**) over-represented biofunctions in omic datasets derived from 18-month-old Tg2576 mice. In (**A**), 45 out of 54 molecules present in cell death category are related to apoptosis (See Table S4).

To obtain a more detailed description of the proteogenomic modulation in the Tg2576 OBs, differential datasets were analyzed for higher-level organization of genes and proteins into common biological pathways. For that, differential proteomic and transcriptomic datasets were merged and functionally analyzed across specific biological functions using the Ingenuity Pathway Analysis (IPA) software (V. 36601845, Release Date: 22 June 2017, Ingenuity Systems^®^, Redwood City, CA, USA). Some statistically over-represented processes were directly relevant to cell movement (*p*-value: 0.0003), cell survival (*p*-value: 0.00015), and cell death (*p*-value: 1.08 × 10^−6^) (Figure 3A and Table S4). Moreover, molecular clusters involved in learning (*p*-value: 0.004), cognition (*p*-value: 0.0007), behavior (*p*-value: 0.003), and dementia (*p*-value: 0.003) were also significantly represented (Figure 3B and Table S4).

**Figure 4:**
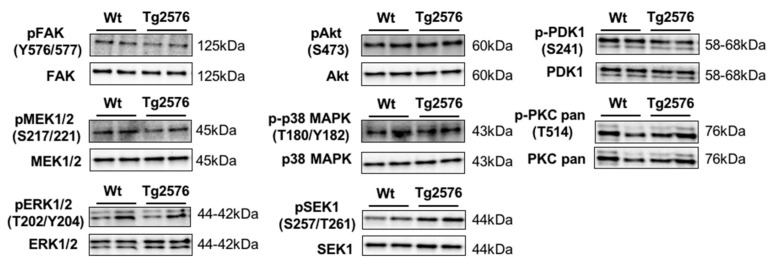
Activation state of specific survival pathways in the OB of aged Tg2576 mice. Levels and residue-specific phosphorylation of focal adhesion kinase (FAK), ERK Activator Kinase 1 (MEK)/extracellular signal-regulated kinase (ERK), protein kinase B (Akt), phosphoinositide-dependent protein kinase 1 (PDK1), protein kinase C (PKC), p38 mitogen-activated protein kinase (p38 MAPK), and mitogen-activated protein kinase kinase 4 (SEK1/MKK4), in the OB of aged Tg2576 mice. Equal loading of the gels was assessed by stain free digitalization. Equal loading control and quantitation values have been included in Table S5.

Although the role of β-amyloid in olfactory deficits detected in Tg2576 mice has been extensively studied [18,19], there is no information about the survival potential of OB neurons in aged Tg2576 mice. To complement our proteogenomic workflow, survival and apoptotic pathways were monitored to analyze the effect of the β-amyloid burden on the viability of the olfactory neurons in aged Tg2576 mice. For that, steady-state levels of survival proteins like Bcl-xL and activated forms of caspase-3, -9, and -12 were measured in protein extracts from Tg2576 OBs at 18 months. The characterization of pro- and anti-apoptotic factors revealed no activation of mitochondrial or endoplasmic reticulum apoptotic routes in aged Tg2576 mice at the level of OB. Subsequent experiments were performed to monitor specific survival pathways at the level of OB. Total and residue-specific phosphorylation of focal adhesion kinase (FAK), protein kinase B (Akt), ERK activator kinase 1 (MEK)/extracellular signal-regulated kinase (ERK), Phosphoinositide-dependent protein kinase 1 (PDK1), protein kinase C (PKC), p38 mitogen-activated protein kinase (p38 MAPK), and mitogen-activated protein kinase kinase 4 (SEK1/MKK4 )were measured in Tg2576 and WT OBs. As shown in Figure 4, no changes in the activation state of this survival panel were observed between transgenic and WT mice at 18 months of age, except a slight increase (non-significant) in the activation state of SEK1/MKK4 kinase (Figure 4).

However, deregulation of MAP kinases (MEK, ERK) and the PDK1/PKC axis has been observed in human OB at advanced AD stages [22,23]. These differences may be partially explained by stage, and species-dependent responses [23] and differences in molecular mechanisms associated to β-amyloidogenesis.

### 2.3. Functional Interactome of the hAPPSw Isoform at the Olfactory Level: Characterization of Potential Hubs by Network-Driven Proteogenomics

**Figure 5:**
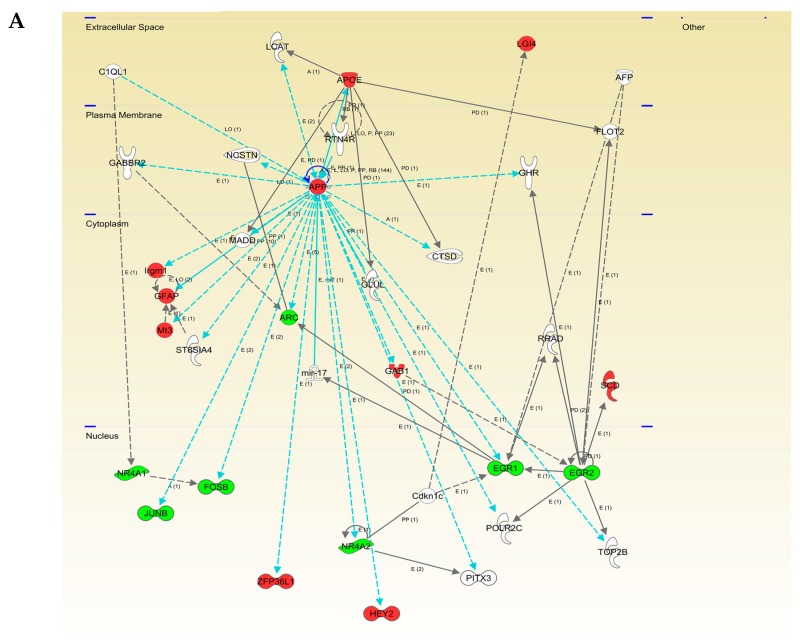

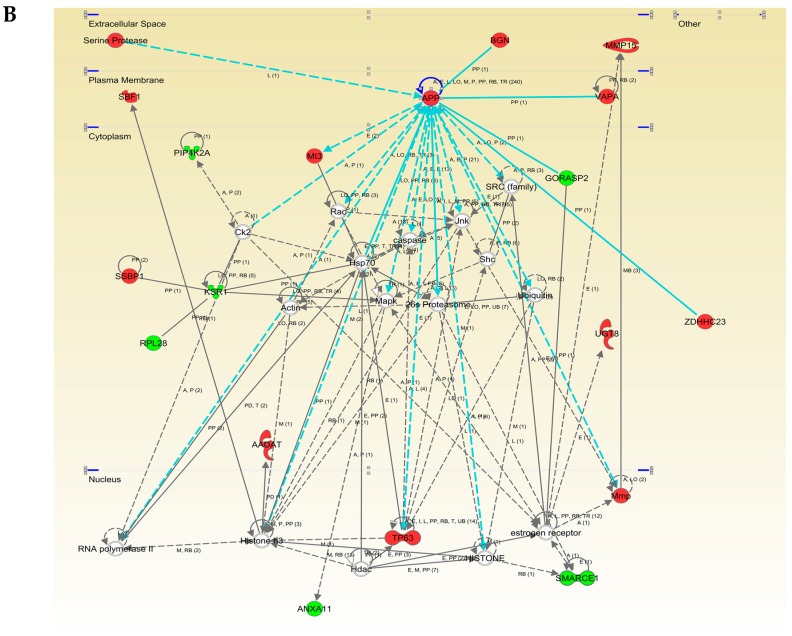
Modulation of APP molecular interaction network in the OB of aged Tg2576 mice. Visual representation of the relationships between differentially expressed genes/proteins and APP functional interactors (blue lines) are shown in both potentially deregulated networks in Tg2576 OBs (**A** and **B**). Continuous lines represent direct interactions, while discontinuous lines correspond to indirect functional interactions. Up-regulated molecules are shown in red, down-regulated molecules in green, and proteins proposed by the software in white. The complete legend of this type of interactomes may be found at http://ingenuity.force.com/ipa/articles/Feature_Description/Legend (accessed on 10 October 2017).

**Figure 6:**
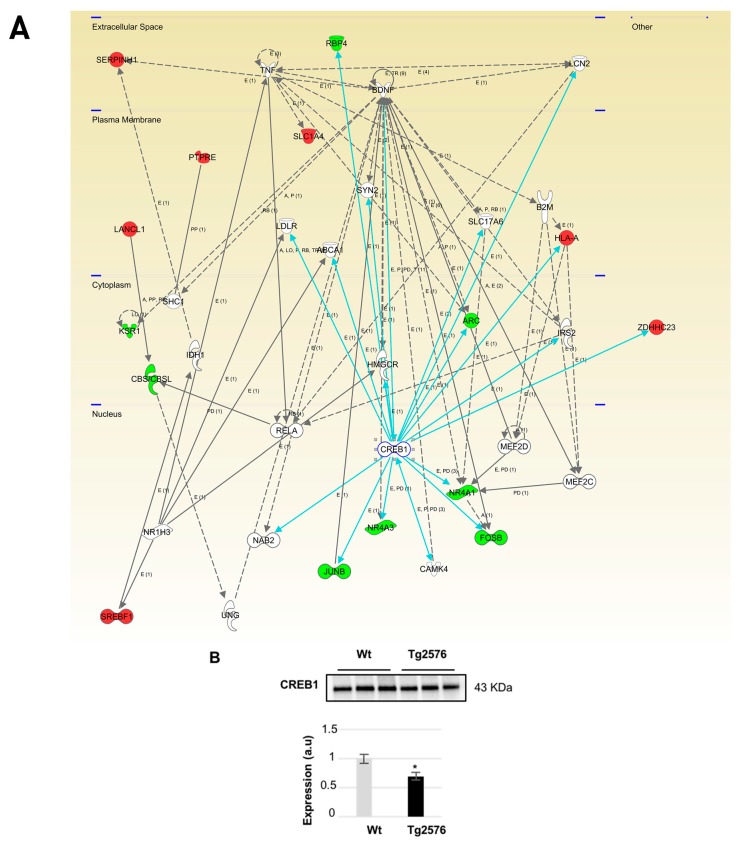
Modulation of CREB1 molecular interactome in the OB of aged Tg2576 mice. Visual representation of the relationships between differential expressed genes/proteins and CREB1 functional interactors (blue lines) are shown in the deregulated network in Tg2576 OBs (**A**); Continuous lines represent direct interactions, while discontinuous lines correspond to indirect functional interactions. Up-regulated molecules are shown in red, down-regulated molecules in green, and molecules proposed by the software in white. The complete legend of this type of interactomes may be found at http://ingenuity.force.com/ipa/articles/Feature_Description/Legend (accessed on 17 October 2017). CREB1 protein levels were down-regulated in the OB of aged Tg2576 mice. * *p* < 0.05 vs. WT group (**B**). Equal loading control and quantitation values have been included in Table S5. a.u., arbitrary units.

To explore the cooperative action among differentially expressed OB genes/proteins in aged Tg2576 mice, we performed molecular interaction networks, merging the olfactory targets that tend to be de-regulated in this model. We consider the discovery of unexpected relationships between apparently unrelated proteins and AD-causing neuropathological substrates as a powerful strategy for the characterization of novel AD causative/susceptibility targets with a central role during olfactory neurodegeneration. Functional interactome maps were generated using IPA software (Figure 5 and Figure 6). We explored whether the hAPPSw isoform, highly expressed in Tg2576 mice, may potentially be interconnected with differential molecular targets detected by our dual-omic approach. As shown in Figure 5, differential functional interactors for APP protein were identified in the OB from aged transgenic mice. The functional APP interactome was composed by targets potentially distributed in different cellular compartments: (i) *APOE*, *SERPINH1*, and *BGN* in the extracellular space; (ii) *VAPA* at the plasma membrane level; (iii) *Irgm1*, *GFAP*, *MT3*, *ARC*, *GAB1*, *ZDHHC23*, and *GORASP2* in the cytoplasm; and (iv) *FOSB*, *JUNB*, *ZFP36L1*, *NR4A2*, *HEY2*, *EGR1*, *TP63*, and *Mmp* in the nuclear compartment (Figure 5).

Moreover, integrative network analysis also allowed us to establish a framework to map interactions between differentially expressed targets and network hubs. According to IPA analysis, CREB1, NFKBIA (IκBα), and NFκB were postulated as potential upstream regulators of part of the differential targets detected in our study (Table 1).

Even though changes in their expression were not detected in our system-wide approaches, the alteration of some of their targets may correspond to a dysregulation in their functionality in the OB of aged Tg2576 mice. For that, subsequent experiments were performed to monitor the protein expression of CREB1 as well as the activation state of IκBα-NFκB p65 complex in the OB of aged Tg2576 mice. CREB1 is a pivotal molecule during synaptic strengthening and memory formation processes [38,39,40]. Protein expression levels of transcription factor CREB1 were significantly reduced in the OB of Tg2576 mice (Figure 6) in parallel with the down-regulation of *FOSB*, *NR4A2*, and *EGR1*, well-known CREB target genes [41] (Figure 6 and Table S3). Our data obtained in aged Tg2576 mice reinforce the direct relationship between a reduction of CREB1 activation and AD pathology [41,42].

### 2.4. An Impairment in the Olfactory IκBα-NFκB p65 Complex Functionality during the Progression of Alzheimer’s Disease (AD) in Tg2576 Mice

**Figure 7:**
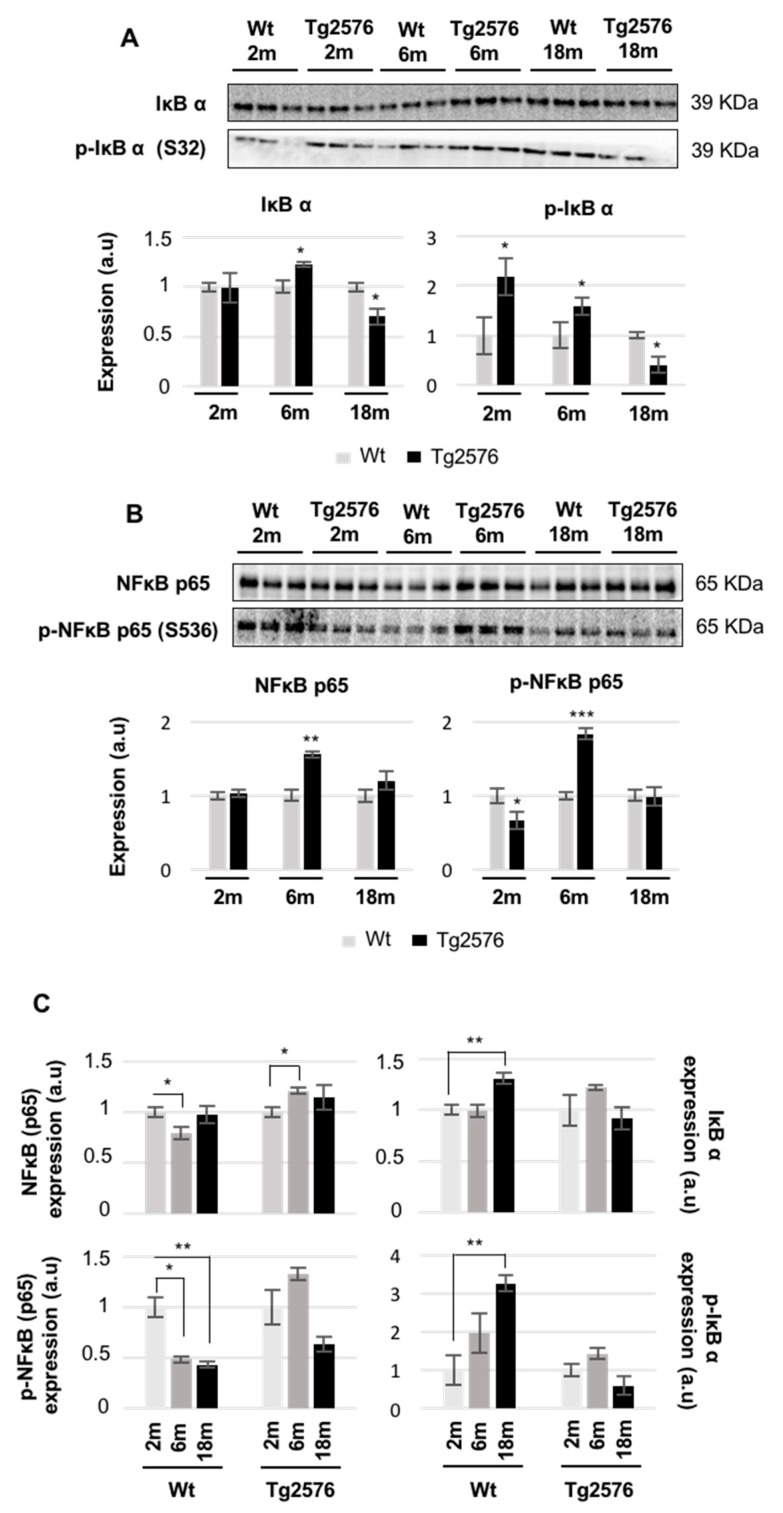
Time-dependent functionality of the OB IκBα-NFκB p65 complex in Tg2576 mice. Levels and residue-specific phosphorylation of the IκBα (**A**); levels and residue-specific phosphorylation of NFκB p65 subunit (**B**); Equal loading of the gels was assessed by stain-free digitalization. Panels show histograms of band densities. Data are presented as mean ± SEM from three independent OB samples per group. * *p* < 0.05 vs. control group; ** *p* < 0.01 vs. control group; *** *p* < 0.001 vs. control group. The expression of the IκBα-NFκB p65 complex was also evaluated during the aging process in WT and Tg2576 mice. * *p* < 0.05 vs. 2-month-old mice; ** *p* < 0.01 vs. 2-month-old mice (**C**). Equal loading control and quantitation values have been included in Table S5. m, month; a.u., arbitrary units.

The nuclear factor NFκB controls the transcription of a wide variety of genes including pro-apoptotic and pro-survival genes, proinflammatory cytokines, antioxidant enzymes, pro-oxidant enzymes, and many others [43]. In general, the formation of a complex of NFκB dimer with a typical member of IkB family prevents the nuclear translocation and gene activation function of NFκB [44]. The phosphorylation of serine 32 of IκBα leads to ubiquitination and proteasomal degradation of IκBα, allowing for the phosphorylation of the NFκB p65 subunit and the enhancement of the p65 transactivation potential [44]. In our study, we monitored the functionality of the IκBα-NFκB p65 complex at three stages of AD: long before (2 months of age), immediately before (6 months), and after (18 months) the appearance of Aβ plaques [9]. At two months of age, the phosphorylated isoforms of IκBα and NFκB p65 were inversely regulated in Tg2576 transgenic mice (Figure 7A,B). However, both phosphorylated proteins were increased at 6 months of age, whereas a specific drop in IκBα levels was detected in 18-month-old Tg2576 mice (Figure 7A,B). Based on these data, it may be hypothesized that APP overproduction induces a transient activation of NFκB in the OB of Tg2576 mice compared to that of WT mice. It has been previously observed that there is an increased NFκB activity in different hippocampal and cortical structures in post-mortem AD brains [45,46,47], probably due to increased oxidative stress, inflammatory reactions, and toxicity of accumulated Aβ peptides [43]. To deepen our understanding of the functional dynamics of the olfactory IκBα-NFκB p65 complexes during the aging process, steady-state levels and phosphorylated isoforms were independently evaluated in WT and Tg2576 mice during aging. For that, protein profiles were quantified in a time-dependent manner. With respect to data obtained at 2 months of age, a specific drop in NFκB p65 protein levels and a progressive decrease in the phosphorylated NFκB p65 subunit were observed in WT mice, whereas an increase in NFκB p65 activity at 6 months was exclusively observed in Tg2576 mice (Figure 7C). In addition, the increase in total and phosphorylated levels of IkB α observed in 18-month-old WT mice, was blocked in aged Tg2576 mice (Figure 7C). Previous reports have shown that NFκB inhibitors prevent Aβ-induced toxicity in vivo and in vitro AD experimental models [48]. To our knowledge, the early impairment in NFκB functionality observed in Tg2576 mice at the level of OB, might open new avenues of targeting the NFκB signaling cascade at the olfactory level, gaining new insight into disease pathogenesis and identifying potential disease modifying agents. However, it is important to note that the phosphoproteome of IκBα-NFκB complexes is highly complicated [44], and many post-translational modifications (PTMs) have been characterized (see http://www.uniprot.org/uniprot/Q04206 (accessed on 10 October 2017), and http://www.uniprot.org/uniprot/P25963 (accessed on 10 October 2017)), but it is still unclear how the tangled crosstalk between all PTMs regulates the ability of NFκB proteins to induce or to repress defined target genes. Due to the early olfactory imbalance detected in the IκBα-NFκB p65 complex in Tg2576 mice, additional studies are necessary to decipher the effects of APP-dependent NFκB dysregulation on the OB molecular landscape at early stages of AD pathology to understand the smell impairment that appears during the AD progression in Tg2576 mice.

Although the accumulation of Aβ oligomers in specific OB regions results in impaired neural integrity in specific OB cell layers in Tg2576 mice during AD progression [49], our results indicate that: (i) 1% of the 2.466 quantified proteins are differentially expressed; and (ii) 0.5% of the 20.900 protein-coding genes are differentially modulated in the OB of 18-month-old Tg2576 mice. These data pointed out that AD-related effects on the OB transcriptome and proteome composition at the bulk level are not as massive at RNA and protein levels at they are at old stages. It is important to note that due to technical reasons, only the most abundant OB proteins corresponding to approximately 10% of the mouse proteome were explored. Consequently, alterations other than those reported in this study might also participate in the AD neurodegeneration at the level of the OB in aged Tg2576 mice. Despite the analysis of the OB proteogenome providing a unique window into their biochemistry and dysfunction in aged Tg2576 mice, there are limitations of our study that warrant discussion. The OB is composed by multiple cell types with a tangled connectivity and architecture [50]. In our case, the information about specific-cell types from where proteins and mRNAs originated is lost due to the processing of the bulk OB in our omic strategy. The implementation of novel approaches that allow the exploration of olfactory cell-type specific molecular profiling would complement the output of our nonbiased exploration of the OB transcriptome/proteome, reducing the effect of multiple neuronal populations in the aged Tg2576 OBs.

## 3. Materials and Methods

### 3.1. Materials

The following reagents and materials were used. From Cell Signaling technology (Danvers, MA, USA): anti-APP (ref. 2450), anti-FAK (ref. 3285), anti-phospho-FAK (Y576/577) (ref. 3281), anti-Akt (ref. 4685), anti-phospho-Akt (S473) (ref. 4060), anti-PDK1 (ref. 3062), anti-phospho-PDK1 (S241) (ref. 3061), anti-phospho-PKC pan (T514) (ref. 9379), anti-p38 MAPK (ref. 9212), anti-phospho-p38 MAPK (T180/Y182) (ref. 9211), anti-MEK1/2 (ref. 9126), anti-phospho-MEK1/2 (S217/221) (ref. 9154), anti-ERK1/2 (ref. 9102), anti-phospho-ERK1/2 (T202/y204) (ref. 4370), anti-CREB (ref. 9104), anti-IκBα (ref. 4814S), anti-phospho-IκBα (S32) (ref. 2859), anti-NFκB p65 (ref. 8242), anti-phospho-NFκB p65 (ref. 3033S), anti-SEK1 (ref. 9152), and anti-phospho-SEK1 (S257/T261) (Ref. 9156). Anti-PKC-pan was from SigmaAldrich (St Louis, MO, USA) (ref. SAB4502356). Electrophoresis reagents were purchased from Bio-Rad (Hercules, CA, USA) and trypsin from Promega (Madison, WI, USA).

### 3.2. Animals

Female Tg2576 transgenic mice were used [3]. Animals were housed 4–5 per cage with free access to water and food, and they were maintained in a temperature-controlled environment on a 12 h light–dark cycle. The progressive development of AD signs in our colony has been previously described [51]. Animal care procedures were conducted in accordance with the European Community Council Directive (2010/63/EU, 22 September 2010) and approved by the local ethics committee. Twelve aged animals (6 WT and 6 Tg2576 mice of 18 months of age), divided into two sets, were used for proteomics and transcriptomics analysis (3 mice/group/platform).

### 3.3. Olfactory Proteomics

OB protein extraction, protein digestion, and peptide iTRAQ labelling was performed as previously described [20,21,22,23]. Tryptic digests were labelled according to the manufacturer’s instructions with one isobaric amine-reactive tag as follows: Tag113, WT-1; Tag114, WT-2; Tag115, WT-3; Tag116, Tg2576-1; Tag117, Tg2576-2; Tag118, Tg2576-3. After 2 h incubation, the set of labelled samples were pooled and evaporated in a vacuum centrifuge. To increase the proteome coverage, the peptide pool was submitted to cation exchange chromatography using spin Columns (Pierce, Rockford, IL, USA). Twelve fractions were collected (from 10 mM to 150 mM KCl), evaporated under vacuum, and reconstituted into 10 μL of 2% acetonitrile, 0.1% formic acid, and 98% MilliQ-H_2_O prior to mass spectrometric analysis. Peptide mixtures were separated by reverse phase chromatography and analyzed by mass-spectrometry as previously described [20,21,22,23]. The raw MS/MS spectra search were processed using the MaxQuant software (v. 1.5.8.3) [52]. The parameters used were as follows: initial maximum precursor (25 ppm), fragment mass deviations (40 ppm); variable modification (methionine oxidation and N-terminal acetylation) and fixed modification (MMTS); enzyme (trypsin) with a maximum of one missed cleavage; minimum peptide length (7 amino acids); false discovery rate (FDR) for peptide spectrum match (PSM), and protein identification (1%). The frequently observed laboratory contaminants were removed. Protein identification was considered valid with at least one unique or “razor” peptide. The protein quantification was calculated using at least two razor + unique peptides, and statistical significance was calculated by a two-way Student-*t* test (*p* < 0.05). A 1.3-fold change cut-off was used. Proteins with iTRAQ ratios below the low range (0.77) were considered to be down-regulated, whereas those above the high range (1.3) were considered to be upregulated. The Perseus software (version 1.5.6.0) [53] was used for statistical analysis and data visualization. Search results files and MS raw data were deposited to the ProteomeXchange Consortium (Available online: http://proteomecentral.proteomexchange.org; accessed on 20 September 2017) via the PRIDE partner repository [54] with the identifier PXD007795.

### 3.4. OB Transcriptomics

Maxwell^®^ 16 simplyRNA Kit (Promega) was used to extract the OB mitochondrial RNAs (mRNAs) from aged Tg2576 mice and WT littermates. The sense complementary DNA (cDNA) was fragmented and biotinylated using the Affymetrix Clarion S Pico assay (902932; ThermoFisher Scientific, Waltham, MA, USA). Affymetrix mouse Clarion S chips (ThermoFisher Scientific) were used according to the manufacturer protocols. Hybridization, washing, staining, scanning, and data analysis [55] were performed as previously described [34,35]. As in other transcriptomic studies performed in AD brains [56,57], we worked with a *p*-value < 0.01 (without using any method for multiple testing correction). Microarray data files were submitted to the GEO (Gene Expression Omnibus) database and are available under accession number GSE103835.

### 3.5. Bioinformatics

The identification of specifically dysregulated regulatory/metabolic networks was analyzed using QIAGEN’s Ingenuity Pathway Analysis (IPA) (Available online: www.qiagen.com/ingenuity; accessed on 10 October 2017) and STRING software [31]. The IPA software considers signaling pathway/biofunctions according to the calculated *p*-value and reports it hierarchically.

### 3.6. Immunoblotting Analysis

Equal amounts of OB protein (5 μg) were resolved in 4–15% TGX stain-free gels (Bio-Rad). OB proteins derived from murine samples were electrophoretically transferred onto nitrocellulose membranes using a Trans-blot Turbo transfer system (up to 25 V, 7 min) (Bio-Rad). Equal loading of the gels was assessed by stain free digitalization and by Ponceau staining. Western-blotting was performed as previously described [20,21,22,23]. After densitometric analyses (Image Lab Software Version 5.2; Bio-Rad), optical density values were expressed as arbitrary units and normalized to total stain in each gel lane.

## 4. Conclusions

The largely similar OB proteogenome of aged WT and Tg2576 mice suggests that abnormal protein-protein interactions or post-translational modifications, defective intracellular trafficking, or misfolding of proteins could play a pivotal part in driving the neurodegeneration that occurs at the olfactory level in aged Tg2576 mice. Moreover, omics sciences have partially revealed the potential interactome of the hAPPSw isoform at the olfactory level as well as the disruption of the IκBα-NFκB p65 complex during the neurodegenerative process, providing molecular features that may be used as novel olfactory drug target candidates to treat AD.

## Figures and Tables

**Table 1:** Potential upstream regulators of differential targets detected in our study.

Upstream Regulator	Molecule Type	*p*-Value	Target Molecules in OB Omics Dataset
CREB1	transcription regulator	0.00000209	*APOE*, *ARC*, *EGR1*, *EGR2*, *FOSB*, *GNAL*, *HLA-A*, *JUNB*, *NR4A1*, *NR4A2*, *NR4A3*, *RBP4*, *SCD*, *ZDHHC23*
NFKBIA	transcription regulator	0.00916	*BCL2A1*, *CD82*, *HLA-A*, *JUNB*, *MMP15*, *NID1*, *NR4A1*
NFKB1	transcription regulator	0.0000748	*APOE*, *APP*, *BCL2A1*, *CD82*, *EGR1*, *HLA-DMB*, *NR4A1*, *TBX21*
NFκB (complex)	complex	0.00174	*APOE*, *APP*, *BCL2A1*, *CD82*, *EGR1*, *GFAP*, *HLA-A*, *HLA-DMB*, *JUNB*, *KLF3*

## Supplementary Materials

Supplementary materials can be found at www.mdpi.com/1422-0067/18/11/2260/s1.

## Abbreviations

AD	Alzheimer’s disease
APP	Amyloid precursor protein
Aβ	β-amyloid
ERK	Extracellular signal-regulated kinase
FAK	Focal adhesion kinase
OB	Olfactory bulb
MS/MS	Tandem mass-spectrometry
PDK1	Phosphoinositide-dependent protein kinase 1
PKC	Protein kinase C
p38 MAPK	p38 Mitogen-activated protein kinase
WT	Wild-type
