# Supplementary material for: Network-Driven Proteogenomics Unveils an Aging-Related Imbalance in the Olfactory IκBα-NFκB p65 Complex Functionality in Tg2576 Alzheimer’s Disease Mouse Model
Source: Int J Mol Sci. 2017 Oct 27;18(11):2260. doi: 10.3390/ijms18112260 (PMC5713230; doi:10.3390/ijms18112260)
Supplement: Supplementary file 1 [file ijms-18-02260-s001.zip › Table S5. Equal loading control.pdf]

***Network-driven proteogenomics unveils an aging-related imbalance in the olfactory IκB alpha-NFκB p65 complex functionality in Tg2576 Alzheimer´s disease mouse model***

*Maialen Palomino, Mercedes Lachen-Montes, Andrea González-Morales, Karina Ausín, Alberto Pérez-Mediavilla, Joaquín Fernández-Irigoyen, Enrique Santamaría*

# - Table S5 -

# EQUAL LOADING CONTROL & QUANTITATION
# (Figures 2, 4, 6, and 7)

# Figure 2a

## Intensity values corresponding to **APP.** Western Blot

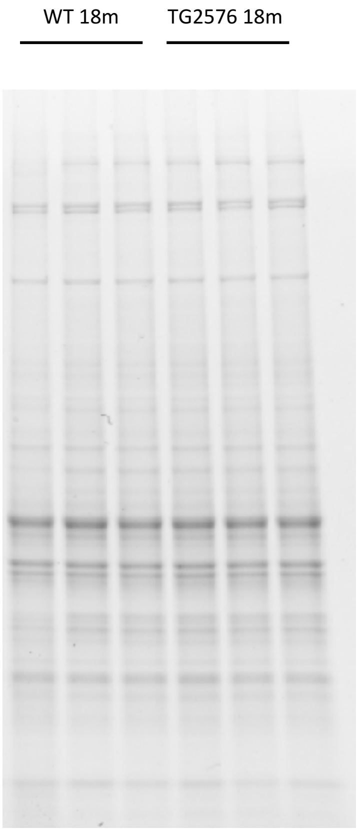


Gel stain free adquisition, 2.5min

| | | Volume (Intensity) | | | | |
|---|---|---|---|---|---|---|
| | **Samples** | **STAIN FREE** | **APP** | **APP/STAIN FREE** | | **Fold of Control** |
| **WT 18 months** | **8673** | 20139803 | 19338 | 0.00096019 | 0.001139948 | 0.84230878 |
| | **8675** | 24335529 | 19316 | 0.00079374 | | 0.69629199 |
| | **8681** | 21763363 | 36256 | 0.00166592 | | 1.46139923 |
| **TG2576 18 months** | **8674** | 23776718 | 236082 | 0.00992912 | | 8.71015654 |
| | **8676** | 20728806 | 176220 | 0.00850121 | | 7.45754533 |
| | **8677** | 21905110 | 155232 | 0.00708657 | | 6.21656969 |

| | | **APP** |
|---|---|---|
| **WT 18 months** | Mean | 1 |
| | SD | 0.40619843 |
| | n | 3 |
| | SEM | 0.23451877 |
| **TG2576 18 months** | Mean | 7.46142385 |
| | SD | 1.24679795 |
| | n | 3 |
| | SEM | 0.71983913 |
| | p value | 0.00365567 |

# Figure 4

## Intensity values corresponding to **pFAK, FAK, pERK and ERK.** Western Blot

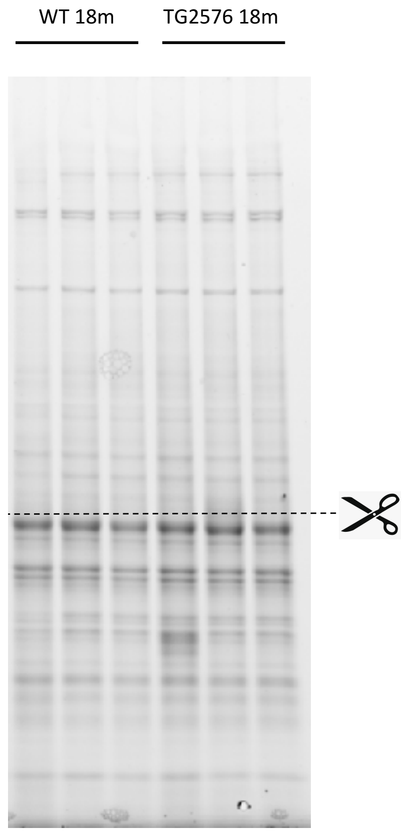


Gel stain free adquisition, 2.5min

| | | Volume (Intensity) | | | | | | | | |
|---|---|---|---|---|---|---|---|---|---|---|
| | Samples | STAIN FREE | pFAK | FAK | pFAK/STAIN FREE | | Fold of Control | FAK/STAIN FREE | | Fold of Control |
| WT 18 months | 8673 | 26500464 | 106146 | 1333496 | 0.00400544 | 0.00366997 | 1.09140998 | 0.05031972 | 0.05741074 | 0.87648627 |
| | 8675 | 29346876 | 137826 | 2059600 | 0.00469645 | | 1.27969668 | 0.07018123 | | 1.22244093 |
| | 8681 | 24558732 | 56682 | 1270454 | 0.00230802 | | 0.62889334 | 0.05173125 | | 0.9010728 |
| TG2576 18 months | 8674 | 37941696 | 107496 | 2751466 | 0.00283319 | | 0.77199295 | 0.07251827 | | 1.26314814 |
| | 8676 | 32297508 | 113148 | 1985918 | 0.0035033 | | 0.95458726 | 0.06148827 | | 1.07102393 |
| | 8677 | 29815812 | 123516 | 2020555 | 0.00414263 | | 1.1287931 | 0.0677679 | | 1.18040465 |

| | | pFAK | FAK |
|---|---|---|---|
| WT 18 months | Mean | 1 | 1 |
| | SD | 0.33489265 | 0.19303134 |
| | n | 3 | 3 |
| | SEM | 0.19335036 | 0.1114467 |
| TG2576 18 months | Mean | 0.9517911 | 1.17152557 |
| | SD | 0.17841651 | 0.09636938 |
| | n | 3 | 3 |
| | SEM | 0.10300882 | 0.05563889 |
| | p value | 0.41986742 | 0.13201595 |

| | | Volume (Intensity) | | | | | | | | |
|---|---|---|---|---|---|---|---|---|---|---|
| | Samples | STAIN FREE | pERK | ERK | pERK/STAIN FREE | | Fold of Control | ERK/STAIN FREE | | Fold of Control |
| WT 18 months | 8673 | 26500464 | 1697472 | 2752902 | 0.06405443 | 0.0735962 | 0.87034967 | 0.10388128 | 0.10214968 | 1.01695153 |
| | 8675 | 29346876 | 3424212 | 3125020 | 0.11668063 | | 1.58541658 | 0.10648561 | | 1.04244681 |
| | 8681 | 24558732 | 983664 | 2359656 | 0.04005353 | | 0.54423374 | 0.09608216 | | 0.94060165 |
| TG2576 18 months | 8674 | 37941696 | 1461474 | 3564108 | 0.03851894 | | 0.52338224 | 0.09393644 | | 0.91959605 |
| | 8676 | 32297508 | 3068406 | 3856176 | 0.09500442 | | 1.29088763 | 0.11939547 | | 1.16882856 |
| | 8677 | 29815812 | 3571164 | 3594798 | 0.11977417 | | 1.62745048 | 0.12056683 | | 1.18029572 |

| | | pERK | ERK |
|---|---|---|---|
| WT 18 months | Mean | 1 | 1 |
| | SD | 0.53256204 | 0.05299646 |
| | n | 3 | 3 |
| | SEM | 0.30747484 | 0.03059752 |
| TG2576 18 months | Mean | 1.14724011 | 1.08957344 |
| | SD | 0.56587775 | 0.14731636 |
| | n | 3 | 3 |
| | SEM | 0.32670967 | 0.08505314 |
| | p value | 0.37964307 | 0.20371266 |

# Figure 4

# Intensity values corresponding to **pAKT, AKT, pP38 and P38.** Western Blot

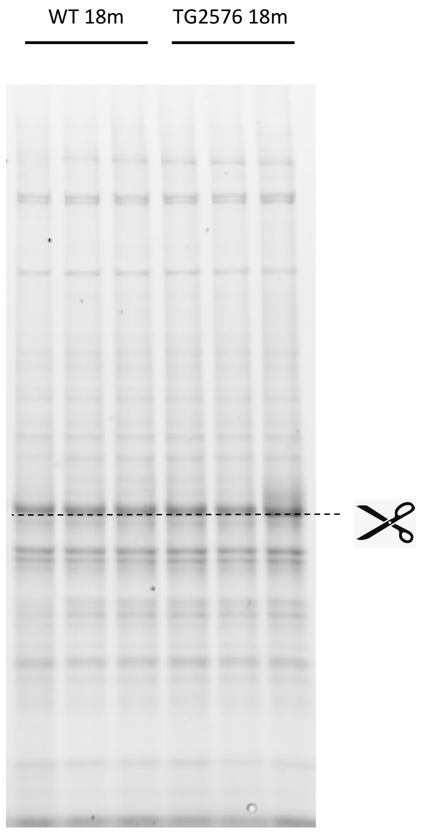


Gel stain free adquisition, 2.5min

| | | Volume (Intensity) | | | | | | | | |
|---|---|---|---|---|---|---|---|---|---|---|
| | **Samples** | **STAIN FREE** | **pAKT** | **AKT** | **pAKT/STAIN FREE** | | **Fold of Control** | **AKT/STAIN FREE** | | **Fold of Control** |
| **WT 18 months** | **8673** | 23178317 | 1378507 | 3248905 | 0.05947399 | 0.08546237 | 0.69590849 | 0.14017001 | 0.17967727 | 0.78012101 |
| | **8675** | 22574958 | 1824551 | 3769220 | 0.0808219 | | 0.94570158 | 0.16696465 | | 0.92924748 |
| | **8681** | 19172586 | 2225769 | 4446068 | 0.11609123 | | 1.35838993 | 0.23189715 | | 1.29063151 |
| **TG2576 18 months** | **8674** | 23416079 | 2043659 | 4333340 | 0.08727588 | | 1.02122001 | 0.18505831 | | 1.02994836 |
| | **8676** | 20415527 | 2173942 | 4236411 | 0.10648474 | | 1.24598386 | 0.20750926 | | 1.15489992 |
| | **8677** | 25993166 | 2719679 | 4152925 | 0.10463054 | | 1.22428781 | 0.15976988 | | 0.88920473 |

| | | **pAKT** | **AKT** |
|---|---|---|---|
| **WT 18 months** | Mean | 1 | 1 |
| | SD | 0.33456188 | 0.26250654 |
| | n | 3 | 3 |
| | SEM | 0.19315939 | 0.15155822 |
| **TG2576 18 months** | Mean | 1.16383056 | 1.02468434 |
| | SD | 0.12397986 | 0.13292579 |
| | n | 3 | 3 |
| | SEM | 0.07157981 | 0.07674474 |
| | p value | 0.24700508 | 0.4468967 |

| | | Volume (Intensity) | | | | | | | | |
|---|---|---|---|---|---|---|---|---|---|---|
| | **Samples** | **STAIN FREE** | **pP38** | **P38** | **pP38/STAIN FREE** | | **Fold of Control** | **P38/STAIN FREE** | | **Fold of Control** |
| **WT 18 months** | **8673** | 23178317 | 772711 | 527098 | 0.03333767 | 0.04580647 | 0.7277939 | 0.022741 | 0.04258387 | 0.53402844 |
| | **8675** | 22574958 | 861517 | 1064100 | 0.03816251 | | 0.8331249 | 0.0471363 | | 1.10690516 |
| | **8681** | 19172586 | 1263842 | 1109600 | 0.06591922 | | 1.43908121 | 0.0578743 | | 1.35906639 |
| **TG2576 18 months** | **8674** | 23416079 | 1017184 | 1218223 | 0.04343955 | | 0.94832797 | 0.05202506 | | 1.22170834 |
| | **8676** | 20415527 | 1005556 | 1172452 | 0.04925447 | | 1.07527334 | 0.05742943 | | 1.34861938 |
| | **8677** | 25993166 | 976106 | 1362775 | 0.03755241 | | 0.81980585 | 0.0524282 | | 1.23117531 |

| | | **pP38** | **P38** |
|---|---|---|---|
| **WT 18 months** | Mean | 1 | 1 |
| | SD | 0.38388525 | 0.4227806 |
| | n | 3 | 3 |
| | SEM | 0.22163625 | 0.2440925 |
| **TG2576 18 months** | Mean | 0.94780239 | 1.26716768 |
| | SD | 0.12773456 | 0.07069788 |
| | n | 3 | 3 |
| | SEM | 0.07374758 | 0.04081744 |
| | p value | 0.42027346 | 0.1940481 |

# Figure 4

# Intensity values corresponding to **pPKC, PKC, pPDK1 and PDK1.** Western Blot

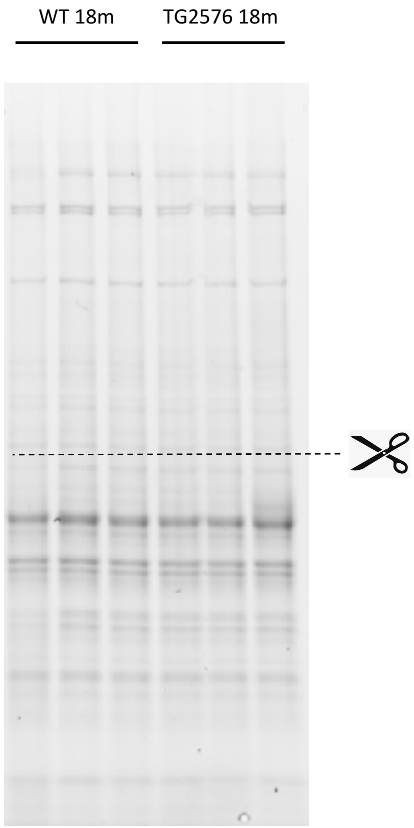


Gel stain free adquisition, 2.5min

| | | Volume (Intensity) | | | | | | | | |
|---|---|---|---|---|---|---|---|---|---|---|
| | **Samples** | **STAIN FREE** | **pPKC pan** | **PKC pan** | **pPKC/STAIN FREE** | | **Fold of Control** | **PKC/STAIN FREE** | | **Fold of Control** |
| **WT 18 months** | **8673** | 27021528 | 1911474 | 535644 | 0.070738931 | 0.09372204 | 0.75477367 | 0.01982286 | 0.02924261 | 0.67787587 |
| | **8675** | 29219508 | 3888630 | 1285200 | 0.13308335 | | 1.41997917 | 0.04398431 | | 1.50411711 |
| | **8681** | 30425328 | 2353212 | 727794 | 0.07734385 | | 0.82524716 | 0.02392066 | | 0.81800703 |
| **TG2576 18 months** | **8674** | 31374720 | 2650914 | 839466 | 0.084492037 | | 0.90151723 | 0.02675613 | | 0.91497048 |
| | **8676** | 26838468 | 5088312 | 1215126 | 0.189590255 | | 2.02289929 | 0.04527554 | | 1.54827259 |
| | **8677** | 35575308 | 4037850 | 931518 | 0.113501477 | | 1.21104356 | 0.0261844 | | 0.89541923 |

| | | **pPKC pan** | **PKC pan** |
|---|---|---|---|
| **WT 18 months** | Mean | 1 | 1 |
| | SD | 0.36541553 | 0.44216482 |
| | n | 3 | 3 |
| | SEM | 0.21097275 | 0.25528398 |
| **TG2576 18 months** | Mean | 1.37848669 | 1.1195541 |
| | SD | 0.57913931 | 0.37140977 |
| | n | 3 | 3 |
| | SEM | 0.33436623 | 0.21443353 |
| | p value | 0.20092239 | 0.36926717 |

| | | Volume (Intensity) | | | | | | | | |
|---|---|---|---|---|---|---|---|---|---|---|
| | **Samples** | **STAIN FREE** | **pPDK1** | **PDK1** | **pPDK1/STAIN FREE** | | **Fold of Control** | **PDK1/STAIN FREE** | | **Fold of Control** |
| **WT 18 months** | **8673** | 27021528 | 2679534 | 462672 | 0.099162934 | 0.14164278 | 0.70009167 | 0.01712235 | 0.02620342 | 0.65343935 |
| | **8675** | 29219508 | 5007744 | 945540 | 0.171383584 | | 1.20997045 | 0.03235989 | | 1.23494886 |
| | **8681** | 30425328 | 4697118 | 886230 | 0.154381836 | | 1.08993788 | 0.02912803 | | 1.11161179 |
| **TG2576 18 months** | **8674** | 31374720 | 3793500 | 741366 | 0.120909446 | | 0.85362234 | 0.02362941 | | 0.9017679 |
| | **8676** | 26838468 | 3610962 | 690822 | 0.134544267 | | 0.94988437 | 0.02573999 | | 0.98231416 |
| | **8677** | 35575308 | 3336282 | 520956 | 0.093780832 | | 0.66209396 | 0.01464375 | | 0.55884883 |

| | | **pPDK1** | **PDK1** |
|---|---|---|---|
| **WT 18 months** | Mean | 1 | 1 |
| | SD | 0.26657214 | 0.30640043 |
| | n | 3 | 3 |
| | SEM | 0.1539055 | 0.17690037 |
| **TG2576 18 months** | Mean | 0.82186689 | 0.8143103 |
| | SD | 0.14649961 | 0.22487185 |
| | n | 3 | 3 |
| | SEM | 0.08458159 | 0.12982982 |
| | p value | 0.19139064 | 0.22452876 |

# Figure 4

# Intensity values corresponding to **pMEK and MEK.** Western Blot

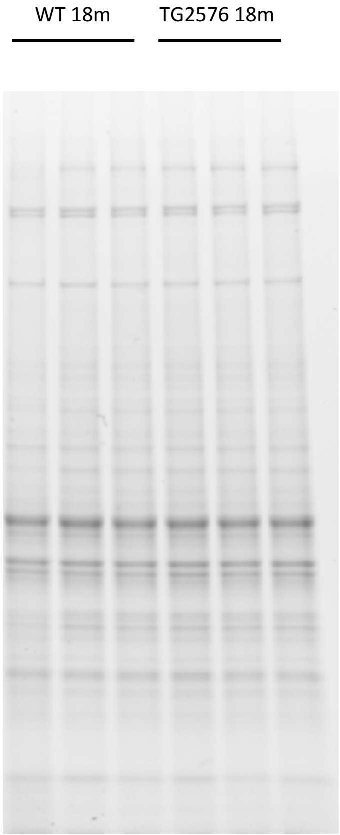


Gel stain free adquisition, 2.5min

| | | Volume (Intensity) | | | | | | | | |
|---|---|---|---|---|---|---|---|---|---|---|
| | **Samples** | **STAIN FREE** | **pMEK** | **MEK** | **pMEK/STAIN FREE** | | **Fold of Control** | **MEK/STAIN FREE** | | **Fold of Control** |
| **WT 18 months** | **8673** | 20139803 | 124146 | 2344410 | 0.006164211 | 0.00864722 | 0.71285479 | 0.1164068 | 0.13934368 | 0.83539347 |
| | **8675** | 24335529 | 217892 | 3457430 | 0.008953658 | | 1.0354379 | 0.14207334 | | 1.01958945 |
| | **8681** | 21763363 | 235562 | 3472364 | 0.010823787 | | 1.25170731 | 0.15955089 | | 1.14501708 |
| **TG2576 18 months** | **8674** | 23776718 | 165756 | 3835644 | 0.006971357 | | 0.80619651 | 0.16131932 | | 1.15770821 |
| | **8676** | 20728806 | 328187 | 3930967 | 0.015832412 | | 1.8309254 | 0.18963789 | | 1.36093643 |
| | **8677** | 21905110 | 310935 | 3412077 | 0.014194633 | | 1.64152588 | 0.15576626 | | 1.11785664 |

| | | **pMEK** | **MEK** |
|---|---|---|---|
| **WT 18 months** | Mean | 1 | 1 |
| | SD | 0.27116857 | 0.15573858 |
| | n | 3 | 3 |
| | SEM | 0.15655925 | 0.08991571 |
| **TG2576 18 months** | Mean | 1.42621593 | 1.21216709 |
| | SD | 0.54523949 | 0.13036975 |
| | n | 3 | 3 |
| | SEM | 0.31479417 | 0.07526901 |
| | p value | 0.15697984 | 0.07343564 |

# Figure 4

# Intensity values corresponding to **pSEK and SEK.** Western Blot

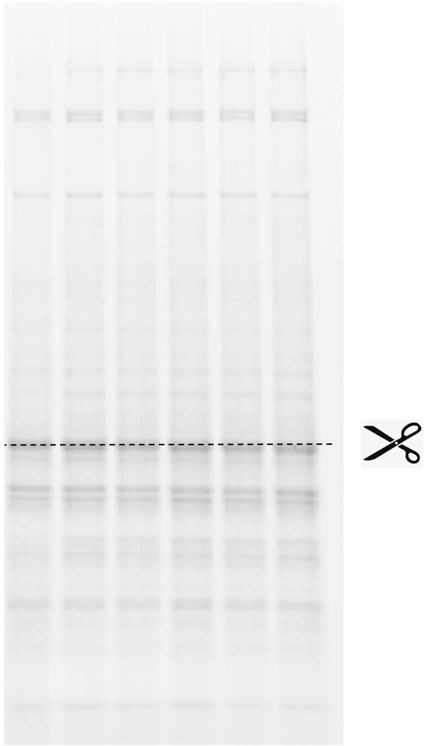


Gel stain free adquisition, 2.5min

| | | Volume (Intensity) | | | | | | | | |
|---|---|---|---|---|---|---|---|---|---|---|
| | **Samples** | **STAIN FREE** | **pSEK** | **SEK** | **pSEK/STAIN FREE** | | **Fold of Control** | **SEK/STAIN FREE** | | **Fold of Control** |
| **WT 18 months** | **8673** | 15393060 | 411825 | 1565118 | 0.02675394 | 0.03171491 | 0.84357621 | 0.10167686 | 0.12173493 | 0.83523163 |
| | **8675** | 18148428 | 706914 | 2354004 | 0.038951803 | | 1.22818602 | 0.129708424 | | 1.06549886 |
| | **8681** | 16967304 | 499500 | 2270556 | 0.029438973 | | 0.92823776 | 0.133819492 | | 1.09926951 |
| **TG2576 18 months** | **8674** | 18114156 | 661820 | 2843802 | 0.036536066 | | 1.15201561 | 0.156993348 | | 1.28963276 |
| | **8676** | 18674388 | 760986 | 2439090 | 0.040750251 | | 1.28489272 | 0.130611509 | | 1.07291732 |
| | **8677** | 19698408 | 995346 | 2917480 | 0.050529261 | | 1.59323386 | 0.1481074 | | 1.21663853 |

| | | **pSEK** | **SEK** |
|---|---|---|---|
| **WT 18 months** | Mean | 1 | 1 |
| | SD | 0.20209785 | 0.14368917 |
| | n | 3 | 3 |
| | SEM | 0.11668125 | 0.08295898 |
| **TG2576 18 months** | Mean | 1.34338073 | 1.19306287 |
| | SD | 0.22634933 | 0.11026448 |
| | n | 3 | 3 |
| | SEM | 0.13068285 | 0.06366123 |
| | p value | 0.0612272 | 0.07168272 |

**Figure 6b** Intensity values corresponding to **CREB1**. Western Blot

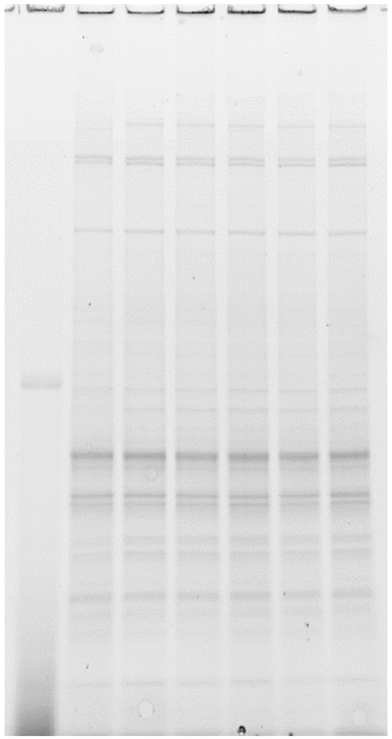

Gel stain free adquisition, 2.5min

| | | Volume (Intensity) | | | | |
|---|---|---|---|---|---|---|
| | **Nombre Muestras** | **STAIN FREE** | **CREB1** | **CREB1/STAIN FREE** | | **Fold of Control** |
| **WT 18months** | **8524** | 21550860 | 3301041 | 0.153174444 | 0.17193765 | 0.89087202 |
| | **8527** | 24729328 | 4096548 | 0.165655452 | | 0.96346233 |
| | **8528** | 22717491 | 4474961 | 0.196983065 | | 1.14566566 |
| **TG2576 18months** | **8522** | 29187591 | 3464076 | 0.118683176 | | 0.69026867 |
| | **8526** | 24587151 | 3475925 | 0.141371605 | | 0.82222598 |
| | **8529** | 26360776 | 2638950 | 0.100108965 | | 0.58223992 |

| | | **CREB1** |
|---|---|---|
| **WT 18months** | Fold of control | 1 |
| | SD | 0.13126767 |
| | n | 3 |
| | SEM | 0.07578742 |
| **TG2576 18months** | Mean | 0.69824486 |
| | SD | 0.12019169 |
| | n | 3 |
| | SEM | 0.0693927 |
| | p value | 0.02146972 |

# Figure 7a

# Intensity values corresponding to **pIKBA and IKBA** Western Blot

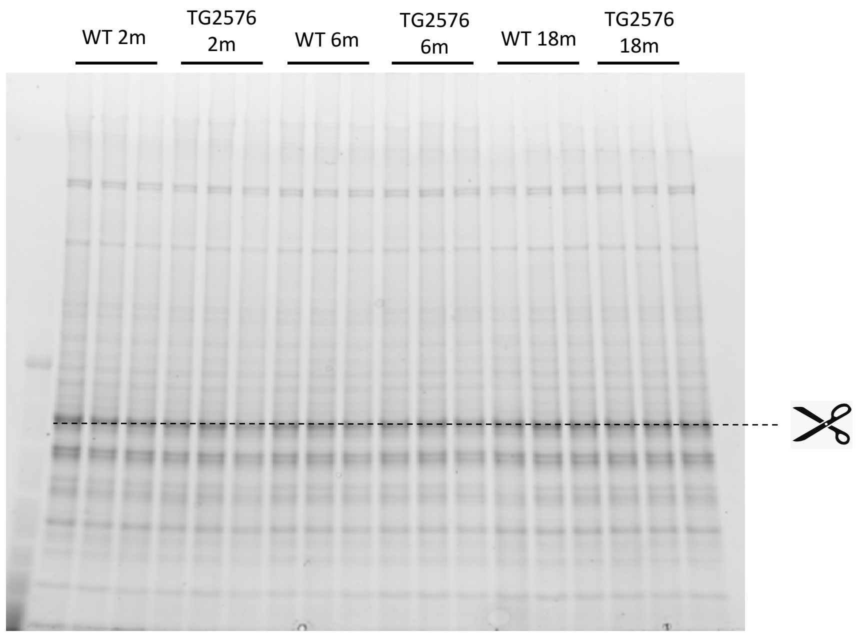


Gel stain free adquisition, 2.5min

| | | p-IkBA | IkBA |
|---|---|---|---|
| **WT 2 months** | Mean | 1 | 1 |
| | SD | 0.65262192 | 0.08335147 |
| | n | 3 | 3 |
| | SEM | 0.37679144 | 0.048123 |
| **TG2576 2 months** | Mean | 2.19249666 | 0.9980587 |
| | SD | 0.64472337 | 0.2570598 |
| | n | 3 | 3 |
| | SEM | 0.37223121 | 0.14841355 |
| | p value | 0.04375552 | 0.49551295 |
| **WT 6 months** | Mean | 1 | 1 |
| | SD | 0.45111096 | 0.10778573 |
| | n | 3 | 3 |
| | SEM | 0.26044903 | 0.06223012 |
| **TG2576 6 months** | Mean | 1.59864948 | 1.23524982 |
| | SD | 0.30575573 | 0.04381985 |
| | n | 3 | 3 |
| | SEM | 0.17652815 | 0.0252994 |
| | p value | 0.06977989 | 0.02400159 |
| **WT 18 months** | Mean | 1 | 1 |
| | SD | 0.10874566 | 0.07022497 |
| | n | 3 | 3 |
| | SEM | 0.06278434 | 0.04054441 |
| **TG2576 18 months** | Mean | 0.40020269 | 0.70310606 |
| | SD | 0.27621712 | 0.14440944 |
| | n | 3 | 3 |
| | SEM | 0.15947403 | 0.08337483 |
| | p value | 0.0245794 | 0.02586233 |

| | | Volume (Intensity) | | | | | | | | |
|---|---|---|---|---|---|---|---|---|---|---|
| | **Samples** | **STAIN FREE** | **p-IkBA** | **IkBA** | **p-IkBA/STAIN FREE** | | **Fold of Control** | **IkBA/STAIN FREE** | | **Fold of Control** |
| **WT 2 months** | **8673** | 43893078 | 1119316 | 193724 | 0.025500969 | 0.01695647 | 1.50390756 | 0.004413543 | 0.00409938 | 1.07663571 |
| | **8675** | 40825338 | 853758 | 169385 | 0.020912454 | | 1.23330208 | 0.004149016 | | 1.01210728 |
| | **8681** | 34103024 | 151963 | 127395 | 0.004455998 | | 0.26279036 | 0.003735592 | | 0.91125701 |
| **TG2576 2 months** | **8674** | 28208654 | 1361938 | 144514 | 0.048280857 | | 2.84734073 | 0.005123038 | | 1.24970914 |
| | **8676** | 37311060 | 1373991 | 154261 | 0.036825301 | | 2.17175469 | 0.004134458 | | 1.00855583 |
| | **8677** | 34935566 | 923168 | 105393 | 0.026424876 | | 1.55839457 | 0.003016782 | | 0.73591113 |
| **WT 6 months** | **8621** | 30123398 | 563057 | 107787 | 0.018691683 | 0.03336435 | 0.56022922 | 0.003578182 | 0.00406011 | 0.8813007 |
| | **8623** | 29834180 | 1454928 | 124393 | 0.048767152 | | 1.46165459 | 0.004169479 | | 1.02693636 |
| | **8625** | 28122698 | 917762 | 124659 | 0.032634209 | | 0.97811619 | 0.004432683 | | 1.09176294 |
| **TG2576 6 months** | **8622** | 36212252 | 1596120 | 174211 | 0.044076795 | | 1.32107466 | 0.00481083 | | 1.18490009 |
| | **8624** | 41292282 | 2133340 | 212040 | 0.051664376 | | 1.54849052 | 0.0051351 | | 1.26476723 |
| | **8626** | 29655846 | 1906056 | 151240 | 0.064272522 | | 1.92638326 | 0.005099838 | | 1.25608215 |
| **WT 18 months** | **8673** | 34257152 | 2127447 | 188157 | 0.062102273 | 0.05519952 | 1.12505086 | 0.005492488 | 0.00538094 | 1.02073063 |
| | **8675** | 36137012 | 1889703 | 205637 | 0.05229273 | | 0.94734021 | 0.005690482 | | 1.05752597 |
| | **8681** | 36380782 | 1862826 | 180443 | 0.051203572 | | 0.92760893 | 0.004959844 | | 0.92174339 |
| **TG2576 18 months** | **8674** | 35859498 | 1152216 | 167371 | 0.032131404 | | 0.58209565 | 0.004667411 | | 0.86739723 |
| | **8676** | 47914428 | 1418046 | 153729 | 0.029595386 | | 0.53615291 | 0.003208407 | | 0.59625429 |
| | **8677** | 43279606 | 196758 | 150366 | 0.004546206 | | 0.08235951 | 0.003474292 | | 0.64566668 |

# Figure 7b

## Intensity values corresponding to **pNFKB and NFKB** Western Blot

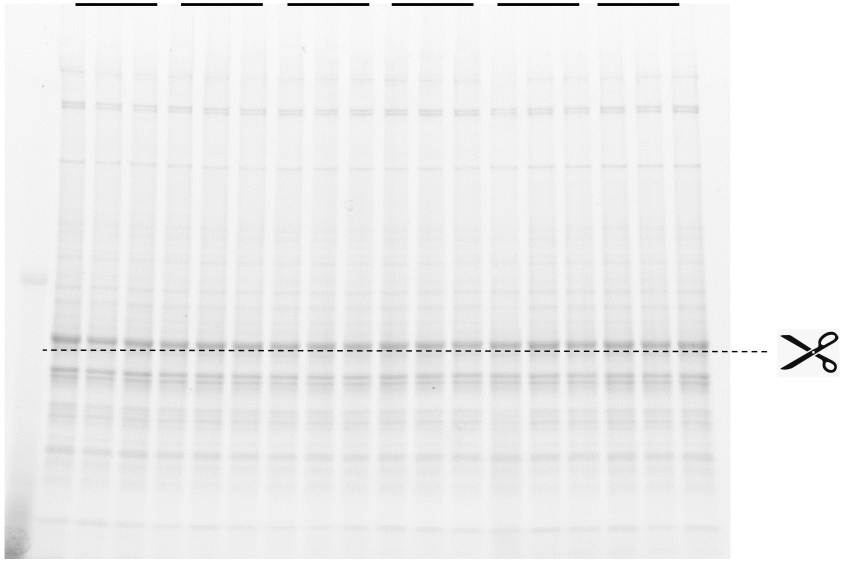


Gel stain free adquisition, 2.5min

| | | pNFKB | NFKB |
|---|---|---|---|
| **WT 2 months** | Mean | 1 | 1 |
| | SD | 0.16840106 | 0.09301045 |
| | n | 3 | 3 |
| | SEM | 0.0972264 | 0.05369961 |
| **TG2576 2 months** | Mean | 0.66944854 | 1.02410997 |
| | SD | 0.20325678 | 0.0841363 |
| | n | 3 | 3 |
| | SEM | 0.11735036 | 0.04857611 |
| | p value | 0.04913662 | 0.37802083 |
| **WT 6 months** | Mean | 1 | 1 |
| | SD | 0.09619447 | 0.13055263 |
| | n | 3 | 3 |
| | SEM | 0.05553791 | 0.0753746 |
| **TG2576 6 months** | Mean | 1.8324013 | 1.56030388 |
| | SD | 0.13389499 | 0.07425869 |
| | n | 3 | 3 |
| | SEM | 0.07730431 | 0.04287328 |
| | p value | 0.00072246 | 0.00316748 |
| **WT 18 months** | Mean | 1 | 1 |
| | SD | 0.13239365 | 0.14367937 |
| | n | 3 | 3 |
| | SEM | 0.07643751 | 0.08295332 |
| **TG2576 18 months** | Mean | 0.98579401 | 1.20493956 |
| | SD | 0.2092376 | 0.22721086 |
| | n | 3 | 3 |
| | SEM | 0.12080339 | 0.13118025 |
| | p value | 0.46322391 | 0.1345078 |

| | | Volume (Intensity) | | | | | | | | |
|---|---|---|---|---|---|---|---|---|---|---|
| | **Samples** | **STAIN FREE** | **pNFKB** | **NFKB** | **pNFKB/STAIN FREE** | | **Fold of Control** | **NFKB/STAIN FREE** | | **Fold of Control** |
| **WT 2 months** | **8673** | 20578656 | 186912 | 2847240 | 0.009082809 | 0.01126938 | 0.80597259 | 0.13835889 | 0.13102285 | 1.05599052 |
| | **8675** | 13561848 | 169362 | 1868202 | 0.012488121 | | 1.10814655 | 0.137754235 | | 1.05137564 |
| | **8681** | 16140456 | 197514 | 1887714 | 0.012237201 | | 1.08588086 | 0.116955432 | | 0.89263384 |
| **TG2576 2 months** | **8674** | 14027616 | 142848 | 1744488 | 0.010183341 | | 0.90362948 | 0.124360975 | | 0.94915484 |
| | **8676** | 13768920 | 83610 | 2011716 | 0.006072372 | | 0.53883828 | 0.146105577 | | 1.11511522 |
| | **8677** | 13568256 | 86526 | 1792080 | 0.006377091 | | 0.56587785 | 0.132078876 | | 1.00805984 |
| **WT 6 months** | **8621** | 13444704 | 67536 | 1425906 | 0.005023242 | 0.00546664 | 0.91889021 | 0.106057076 | 0.10413088 | 1.01849787 |
| | **8623** | 14213376 | 75744 | 1274598 | 0.005329065 | | 0.97483368 | 0.08967595 | | 0.86118501 |
| | **8625** | 14441400 | 87336 | 1684728 | 0.006047613 | | 1.10627611 | 0.116659604 | | 1.12031712 |
| **TG2576 6 months** | **8622** | 14870736 | 158868 | 2435382 | 0.010683264 | | 1.9542652 | 0.163770105 | | 1.57273337 |
| | **8624** | 14267592 | 144594 | 2418066 | 0.010134436 | | 1.85386935 | 0.169479615 | | 1.62756351 |
| | **8626** | 14123520 | 130410 | 2177532 | 0.009233534 | | 1.68906934 | 0.154177712 | | 1.48061475 |
| **WT 18 months** | **8673** | 15393060 | 64440 | 1673892 | 0.004186302 | 0.00486347 | 0.86076426 | 0.108743291 | 0.12768719 | 0.85163821 |
| | **8675** | 18148428 | 99234 | 2638242 | 0.005467912 | | 1.1242817 | 0.145370277 | | 1.13848755 |
| | **8681** | 16967304 | 83754 | 2187900 | 0.0049362 | | 1.01495404 | 0.128948005 | | 1.00987424 |
| **TG2576 18 months** | **8674** | 18114156 | 87282 | 2829168 | 0.004818441 | | 0.99074125 | 0.156185472 | | 1.22318825 |
| | **8676** | 18674388 | 70308 | 2310930 | 0.003764943 | | 0.77412666 | 0.123748634 | | 0.96915464 |
| | **8677** | 19698408 | 114246 | 3577860 | 0.005799758 | | 1.19251413 | 0.181631937 | | 1.42247578 |
